# The trajectory of body image dissatisfaction during pregnancy and postpartum and its relationship to Body-Mass-Index

**DOI:** 10.1371/journal.pone.0309396

**Published:** 2024-08-26

**Authors:** Katja Linde, Franziska Lehnig, Julia Treml, Michaela Nagl, Holger Stepan, Anette Kersting

**Affiliations:** 1 Department of Psychosomatic Medicine and Psychotherapy, University of Leipzig, Leipzig, Germany; 2 Department of Obstetrics, University of Leipzig, Leipzig, Germany; McGill University, CANADA

## Abstract

**Background:**

During pregnancy, women’s bodies undergo rapid body weight and size changes within a relatively short period. Pregnancy may therefore, be associated with an increased vulnerability for developing body image dissatisfaction linked to adverse health outcomes for the mother (e.g., depression, eating disorders) and child (e.g., impaired self-regulation, childhood obesity). The present study aims to examine the prevalence and trajectories of body image dissatisfaction during pregnancy and postpartum and its relationship to pre-pregnancy BMI. This is the first study that investigates prevalence rates of body image dissatisfaction not only ante- but also postpartum, and that compares trajectories of women with normal weight and overweight.

**Methods:**

A prospective longitudinal design with a quantitative approach was applied. Healthy pregnant women (N = 136) answered paper-pencil or online questionnaires at four time points (18th-22nd and 33rd-37th week of gestation, 3 and 6 months postpartum). Body image dissatisfaction was assessed using the German version of the Body Shape Questionnaire (BSQ) and the Eating Disorder Examination Questionnaire (EDE-Q). Both questionnaires are considered reliable and valid measures of several aspects of body image, and the BSQ allows for calculating prevalence rates by providing cut-off values. Using not just one but two body image questionnaires, trajectories of body image dissatisfaction can be compared. Pre-pregnancy BMI was assessed retrospectively via self-reported weight and height.

**Results:**

The proportion of women reporting elevated levels of body image dissatisfaction was 6.6% (n = 9) in the second trimester, 2.9% (n = 4) in the third trimester, 11.0% (n = 15) three months postpartum, and 10.3% (n = 14) six months postpartum. Repeated measures ANOVA revealed that body image dissatisfaction significantly decreased from pre-pregnancy to pregnancy, remained stable during pregnancy, and returned to pre-pregnancy levels three to six months postpartum. Mixed between-within ANOVA showed that the overweight/obese group reported significantly higher levels of body image dissatisfaction at each measurement point except during the third trimester than women in the normal weight group. Significant but small interaction effects between time and pre-pregnancy BMI were found.

**Conclusions:**

The results revealed that approximately every tenth woman is affected by body image dissatisfaction after childbirth. Women with a higher BMI level before pregnancy are particularly at risk of experiencing body image dissatisfaction. Healthcare providers should screen for body image dissatisfaction, in particular after childbirth, and inform affected women about possible adverse health outcomes and treatment options. Study limitations concern the drop-out rate of 51.4% and the retrospective and self-reported assessment of pre-pregnancy BMI. Future studies should include additional assessment points in the first trimester and more than six months postpartum and try to include a matched control group of non-pregnant women to compare prevalence rates and trajectory of body image dissatisfaction.

## Introduction

Body image is a multidimensional construct referring to an individual’s internal representation of his or her outer appearance [1]. Body image dissatisfaction is part of an attitudinal component of body image, referring to a negative evaluation of one’s figure or specific aspects of the body [2]. During pregnancy and the postpartum period, women’s bodies undergo rapid changes in body weight and size within a relatively short (40-week) period. These unique physical changes will likely contribute to reevaluating women’s body image [3]. There are two opposing theories about the effect of pregnancy on body image dissatisfaction [4]. On the one hand, pregnancy-related physical changes (e.g. weight gain) may contribute to body image dissatisfaction during pregnancy and postpartum [5] due to deviations from the ‘thin ideal’ imposed by the media and most Western societies [1]. On the other hand, the degree of body image dissatisfaction might be unchanged or even reduced during pregnancy, as culturally defined beauty ideals might be less relevant during pregnancy [6]. Until now, only two studies [7,8] used a body image measure that provides cut-off values and therefore allows the estimation of the prevalence of body image dissatisfaction during pregnancy. These studies showed that 34% [7] and around 45% [8] of women suffer from body image dissatisfaction during pregnancy. Studies investigating the prevalence of body image dissatisfaction during the postpartum period using body image questionnaires that provide cut-off values have been lacking. Therefore, more studies are needed that use a body image measure with cut-off values and several measurement points during pregnancy and postpartum.

Some studies investigating the course of body image dissatisfaction indicate that body image dissatisfaction increases during pregnancy and decreases postpartum [7,9,10]. One study found that body image dissatisfaction is stable during pregnancy and deteriorates postpartum [11]. Other studies indicate that the degree of body image dissatisfaction decreases from pre-pregnancy to pregnancy and increases in the postpartum period [12–16]. Overall, until today, empirical evidence concerning the trajectory of body image dissatisfaction during pregnancy and postpartum is contradictory. Besides others, these inconsistencies may reflect differences in body image measures applied. A study that uses more than one body image measure with adequate psychometric properties would provide the opportunity to compare trajectories and investigate similarities and differences.

Body image dissatisfaction in non-pregnant populations is a robust risk factor for disordered eating behaviors or attitudes (e.g., dieting, unhealthy weight control behaviors, binge eating, emotional eating, shape, and weight concerns) [17–19] and eating disorders like bulimia [18]. Furthermore, it is a meaningful predictor of depression [17,18,20,21] and low self-esteem [18,22] in women. Likewise, body image dissatisfaction has been consistently linked to incident prenatal and postpartum depression in mothers both in cross-sectional and prospective studies [23]. Body image dissatisfaction in pregnant women has also been identified as a risk factor for symptoms of eating disorders, anorexia nervosa, and longing to eat [24]. Furthermore, body image dissatisfaction appears to directly increase the risk of excessive gestational weight gain, which is defined as a weight gain that goes beyond gestational guidelines [25]. Excessive gestational weight gain itself can result in poorer birth outcomes due to increased maternal and fetal complications, like pregnancy-induced hypertension, cesarean section/assisted delivery, and higher prevalence and risk of birthweight over 4500 g [26]. Besides this, body image dissatisfaction has been linked to a reduced breastfeeding duration [27]. Furthermore, a recent review [28] summarizes the complex association between maternal body dissatisfaction and child obesity risk. According to the conceptual model developed by the authors, maternal body dissatisfaction affects maternal psychopathology, maternal eating behavior as well as mother-child feeding interactions, and the mother-child relationship quality during early parenthood. These factors might harm the child’s self-regulation capacities and attachment security during early childhood, which in turn might lead to a higher risk of childhood obesity. Childhood obesity is a severe public health problem worldwide. It is associated with cardiovascular disease risk, diabetes mellitus, obstructive sleep apnea, psychosocial disturbances, impaired quality of life, and shorter life expectancy [29,30].

Regarding the negative impact of body image dissatisfaction on maternal and fetal health outcomes, identifying risk factors is crucial to derive effective intervention strategies. Besides other factors such as exercise, stress, or social support, some studies indicate that a higher pre-pregnancy body mass index (BMI) might be a risk factor for body image dissatisfaction. Higher levels of pre-pregnancy BMI were related to stronger levels of body image dissatisfaction [6,31], drive for thinness [6] and feeling fat [5] during pregnancy. In contrast, one study found no significant association between body image dissatisfaction and pre-pregnancy BMI [32]. To further verify the role of pre-pregnancy BMI as a risk factor for body image dissatisfaction not only during pregnancy but also in the postpartum period, more research is needed. Until now, only one study [13] investigated the association of pre-pregnancy BMI and body image dissatisfaction postpartum. The study found that postpartum body image dissatisfaction was significantly related to higher levels of pre-pregnancy BMI. Relatedly, a prospective study [16] revealed that eating behavior influences the trajectory of body image dissatisfaction during pregnancy and postpartum. While body image dissatisfaction was reduced during pregnancy and returned to previous levels after delivery in each group of women with normal weight, subthreshold eating disorders (not meeting diagnostic criteria for an eating disorder), and eating disorders, the amelioration of body image dissatisfaction was especially pronounced in women with eating disorders. According to these findings, changes in body image dissatisfaction over pregnancy and postpartum might also be different in women with high or low pre-pregnancy BMI. Therefore, a study is needed that compares the trajectories in body image dissatisfaction of women with high and low pre-pregnancy BMI levels throughout pregnancy and postpartum.

### Aims

The first aim was to estimate the prevalence of women suffering from body image dissatisfaction during pregnancy and postpartum. The second aim was to investigate changes in body image dissatisfaction (body image dissatisfaction, weight concern, shape concern) during pregnancy and postpartum. The third aim was to examine the influence of pre-pregnancy BMI on the level and course of body image dissatisfaction during pregnancy and postpartum by analyzing women with normal weight and women who were overweight/obese before pregnancy separately.

## Materials and methods

### Participants and procedure

Participants were recruited at the Department of Obstetrics of the University Hospital of Leipzig (Germany). While waiting for routine prenatal diagnostic appointments, women were personally informed about the study goals and asked for participation by study staff. Recruitment occurred between April 4th, 2018, and December 23rd, 2019. To be included in the study, women had to be pregnant between the 18^th^ and 22nd week of gestation and above 18 years of age. Women with multiple pregnancies and inadequate German reading or writing skills to answer the questionnaires were excluded from the study. Women who agreed to participate were given study information verbally by study staff and a three-page information sheet covering study goals and procedures, potential risks, rights, and data privacy information. No additional steps were taken for ensuring comprehension among participants with varied educational backgrounds. Afterward, eligible women subscribed to the informed consent sheet and received a paper-pencil version of the first questionnaire or a link for the online version according to their choice. Data were collected at four assessment points: T1 (second trimester: 18^th^-22^nd^ week of gestation), T2 (third trimester: 33^rd^-37^th^ week of gestation), T3 (3 months postpartum), and T4 (6 months postpartum). Non-responders were contacted by email or postal mail up to two times within three weeks. The study was conducted according to the Declaration of Helsinki and was approved by the Ethics Committee of the Medical Faculty of the University of Leipzig (reference number: 422/17-ek, 14.11.2017). The current study is an extension of the study by Linde et al. (2022) [33]. It is based on the same sample of pregnant women who were recontacted and asked for study participation three and six months after delivery.

### Measures

Sociodemographic and health-related variables were measured using self-generated questions. Variables included maternal age, partnership, school education, employment status, household income, and parity. The occurrence of current physical disorders was measured with a dichotomous self-generated item [‘Do you currently suffer from any kind of physical disorders?’].

Body image dissatisfaction was measured using the German version of the Body Shape Questionnaire [BSQ [34,35]] and the Weight and Shape Concern subscales of the German version of the Eating Disorder Examination-Questionnaire [EDE-Q; [36,37]]. Both questionnaires were reliable and valid measures of body image [38]. The BSQ consists of 34 items; the total sum score ranges from 34 to 204, with higher scores indicating higher body shape dissatisfaction. The reliability, factorial, and convergent validity of the BSQ have been demonstrated [39]. The reliability coefficients of the BSQ sum score in the present sample ranged between.96 ≤ α≤ .97. They are comparable to reliability coefficients reported in a German validation study (.94 ≤ α ≤ .96) [40]. A cut-off of > 100 has been considered to indicate significantly elevated levels of body dissatisfaction in a representative sample of women [40]. The EDE-Q’s Weight and Shape Concern subscales consist of five and eight items, respectively. Subscale means were calculated, with higher scores indicating higher weight and shape concern. Both subscales have been shown to have good internal consistency [41]. The reliability coefficients of the Weight and Shape Concern subscales in the present sample ranged between.78 ≤ α≤ .83 and.89 ≤ α≤ .92. These coefficients are comparable to those reported in a German validation study (weight conern:.69 ≤ α≤ .86; shape concern:.85 ≤ α≤ .93) [41]. The BSQ was assessed at T1-T4, while both subscales of the EDE-Q were additionally assessed in the second trimester to assess weight and shape concerns retrospectively for the time before pregnancy [‘Please answer the following questions concerning your body image before getting pregnant.’]. This additional measurement point is conceptualized as T0.

Pre-pregnancy BMI was calculated from self-reported height and retrospectively reported pre-pregnancy weight (BMI = weight (kg)/height (m)^2^). As there was no possibility of assessing the weight before women got pregnant, they were asked to estimate their pre-pregnancy weight during the T1 assessment (18^th^-22^nd^ week of gestation). The degree of certainty of retrospective weight assessment was assessed with a single item using a 10-point response scale [range 1 ’not sure at all’ to 10 ’extremely sure’]. The certainty of pre-pregnancy weight assessment was high, as most of the sample (77.8%) chose a value between eight and ten, and the median was 9. According to the BMI classification of the World Health Organization [42], pre-pregnancy BMI was categorized into four subgroups: underweight (< 18.5 kg/m^2^), normal weight (18.5–24.9 kg/m^2^), overweight (25–29.9 kg/m^2^), and obese [≥ 30 kg/m^2^]. BMI is supposed to indicate the risk of disease [42]. Extreme underweight is associated with poor physical performance, lethargy, and even death. In contrast, overweight and, in particular, obesity increases the risk of coronary heart disease, stroke, diabetes mellitus, cancer, respiratory symptoms, low quality of life, and body pain [42–44].

### Statistical analyses

Data analyses were performed using the Statistical Package for Social Sciences, version 29.0 (IBM^®^ SPSS^®^), and the significance level was set to 0.05.

Before primary analyses, the number and percentages of missing values were calculated for pre-pregnancy BMI and the three body image measures at each assessment occasion (T0-T4). There was one missing value for EDE-Q Weight Concern at T1 (0.7%) and one for EDE-Q Weight Concern at T4 (0.7%). All other variables had zero missing values. Due to the small number of missing values, missing values for EDE-Q Weight Concern at T1 and T4 were imputed by the mean. The number and percentages of study drop-outs from T1 to T4 were calculated. Study drop-outs were not imputed. Differences between study drop-outs and completers were analyzed using independent t-tests for continuous variables or X^2^-tests for categorical variables. Outliers were defined as values more extensive than three standard deviations below or above the mean. Outliers were replaced by values that show less pronounced deviation from the mean (Winsorizing: new value = mean ± 3SD).

Regarding the first aim, the prevalence of body image dissatisfaction was estimated by calculating the percentage of women scoring above the BSQ’s cut-off value for each measurement point (T1-T4).

Regarding the second aim, differences across the time points for each outcome measure were examined via one-way repeated measures analyses of variance (ANOVA). Within-subject factors were BSQ (T1-T4), EDE-Q Weight (T0-T4), and Shape Concern (T0-T4). The prerequisite of normality within groups and the absence of outliers were checked. Furthermore, the prerequisite of homogeneity of variances (sphericity) was checked using Mauchly’s test. The Huynh-Feldt Epsilon (HFE) adjustment was used to correct for violations of sphericity. Bonferroni post hoc comparisons were conducted to detect mean differences between time points. The Cochrane Q-test was used to analyze if the proportion of women scoring above the cut-off value of the BSQ changes significantly over time. (T0-T4).

Regarding the third aim, differences across the time points for each of the three outcome measures in relationship to pre-pregnancy BMI were examined using a mixed between-within ANOVA. Within-subject factors were BSQ (T1-T4), EDE-Q Weight (T0-T4), and Shape Concern (T0-T4). The between-subject factor was pre-pregnancy BMI [normal weight; overweight/obese]. Normality within groups, outliers, and sphericity was checked. Again, the Huynh-Feldt Epsilon (HFE) adjustment was used to correct violations of sphericity. Levene’s test (p >.05) and Box’s test (p >.025) were used to assess the homogeneity of error variances and covariances. The main effects of time, group, and interaction were analyzed. Bonferroni post hoc comparisons were conducted to detect mean differences between time points in case of a significant interaction.

For main and interaction effects, eta-squared (η^2^) effect sizes were calculated. An effect size of 0.01 indicates a small effect, an effect size of 0.06 a medium, and 0.14 a large effect. A large effect size for the main effect of time would mean that there are strong differences in the degree of body image dissatisfaction throughout pregnancy and postpartum. A large effect size for the main effect of group would mean that there are strong differences in the degree of body image dissatisfaction between women with normal weight and women with overweight/obesity. For mean differences between two measurement points, Cohen’s d_RM_ was calculated. An effect size of 0.20 indicates a small effect, an effect size of 0.50 a medium, and 0.80a large effect. A large effect size for the difference between T0 and T1 would imply, for example, a large and meaningful change in body image dissatisfaction from pre-pregnancy (T0) to the second trimester (T1) of pregnancy.

## Results

**Fig 1:**
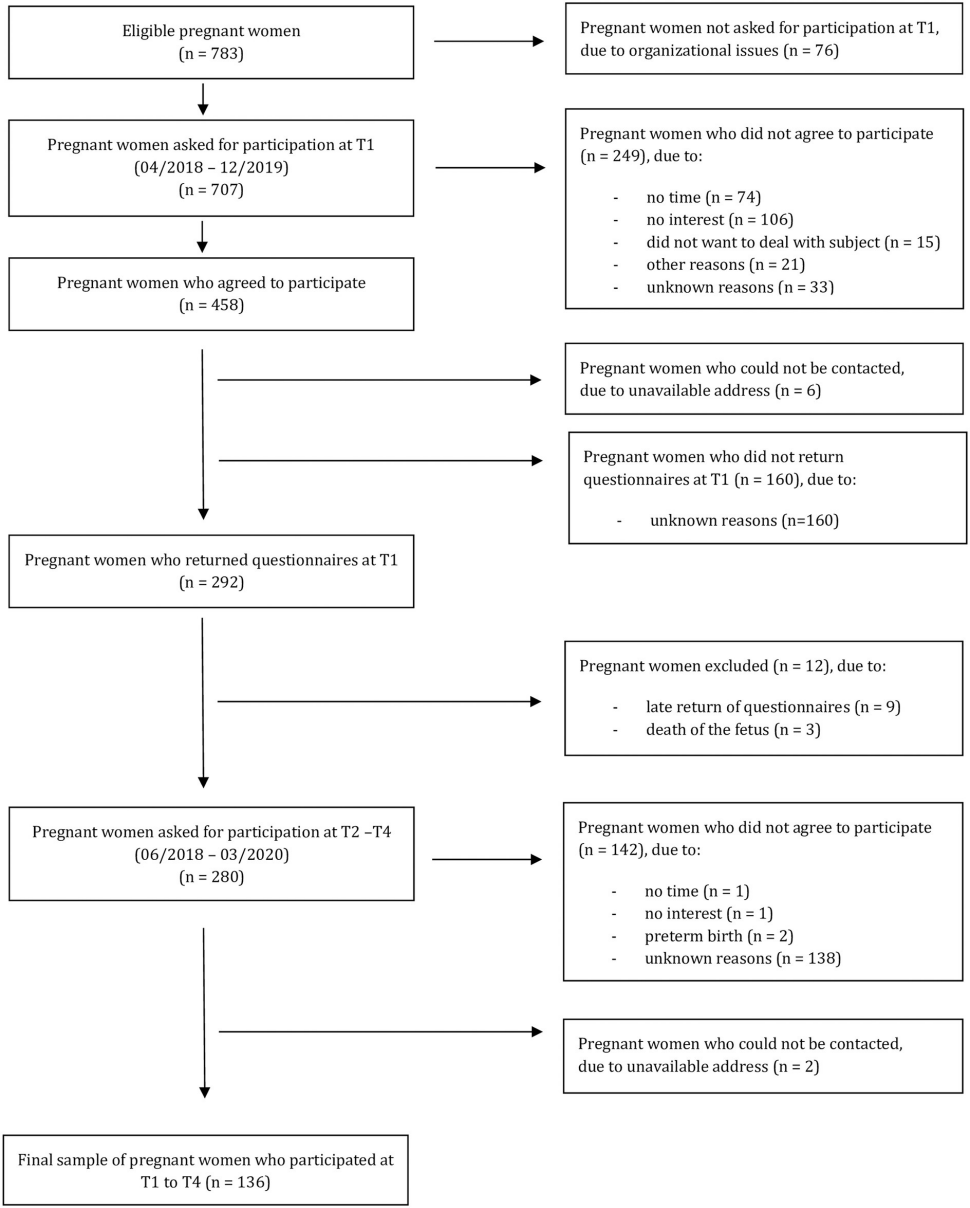
Flow diagram.

A total of 783 women were eligible for study entry, and N = 280 completed T1, 222 (80.1%) T2, 181 (65.3%) T3, and 142 (51.3%) T4 assessment (see Fig 1 for participant flow, drop-out rate: 50.7%). A final sample of N = 136 women showed complete data from T1 to T4. Drop-out analyses revealed significant differences for the BSQ [t(260.58) = 3.16, p = .002], the EDE-Q Weight and Shape Concern subscales [t(268.38) = 2.30, p = .02; t(268.24) = 2.29, p = .02], BMI [t(263.70) = 2.66, p = .01], and school education [χ^2^(2) = 11.03, p = .004]. Women who dropped out showed higher body image dissatisfaction, a higher BMI, and a lower educational level at T1.

The number of outliers ranged between one and three. After Winsorizing, no extreme outliers were detected in box plots. The normality assumption was violated for the outcome variables, as indicated by the Shapiro-Wilk test. Due to the overall sample size greater than 100 and subgroup sample sizes greater than 30, repeated measure ANOVA and between-within ANOVA were considered robust to violations of the assumption of normal distribution. The Greenhouse-Geisser adjustment was used to correct violations of sphericity. Concerning the between-within ANOVA, the assumption of homogeneity of error variances was violated for the EDE-Q Weight and Shape Concern subscales (T1-T4) and partly for the BSQ (T3, T4). The assumption of homogeneity of covariances was violated for the EDE-Q Weight Concern subscale only.

Table 1 shows the sample’s demographic data at the T1 assessment. Participants’ ages ranged from 19 to 45 years. Most women were of German nationality, lived in a partnership, and had a high school education. Around two-thirds of women were full or part-time employed at T1. Nearly half of the women (44.1%) had given birth before. Half of the sample had a household income of less than 3.000€. Mean BMI before pregnancy was in the upper normal range. Nearly half of the women (44.4%) reported suffering from physical disorders, most often thyroid disease, followed by skin, intestinal, and respiratory diseases. The average age of pregnant women, BMI, and distribution to BMI groups were comparable to those of the German population. However, household net income was below the average [45–47].

**Table 1:** Final sample characteristics at T1-T4 assessment, N = 136.

Sociodemographic characteristics	
Age, *M* (*SD*)	31.73 (4.75)
Nationality: German, *n* (%)	131 (96.3)
Partnership, *n* (%)	131 (98.5)
School education	
Low secondary qualification, *n* (%)	2 (1.5)
High secondary qualification, *n* (%)	39 (28.9)
University entrance qualification, *n* (%)	94 (69.6)
Any university degree, *n* (%)	69 (51.1)
Employment status	
Full-time employment, *n* (%)	81 (60.0)
Part-time employment, *n* (%)	22 (16.3)
Employment prohibition due to pregnancy, *n* (%)	13 (9.6)
Others, *n* (%)	19 (14.1)
Household income /month	
≤ 1000 €, *n* (%)	6 (4.5)
1001–2000 €, *n* (%)	35 (26.1)
2001–3000 €, *n* (%)	26 (19.4)
3001–4000 €, *n* (%)	33 (24.6)
4001–5000 €, *n* (%)	24 (17.9)
≥ 5001 €, *n* (%)	10 (7.5)
Weight-related variables	
Pre-pregnancy weight [kg], *M (SD)*	67.28 (14.48)
Pre-pregnancy BMI [kg/m^2^], *M (SD)*	24.02 (4.94)
Underweight (< 18.5), *n* (%)	5 (3.7)
Normal Weight (18.5–24.9), *n* (%)	90 (66.2)
Overweight (25–29.9), *n* (%)	26 (19.1)
Obese (≥ 30), *n* (%)	15 (11.0)

*Notes*. Total sample *N* = 136; *M* = mean; *SD* = standard deviation, *n* = absolute frequency, *%* relative frequency.

### Prevalence of body image dissatisfaction during pregnancy and postpartum

The proportion of women reporting elevated levels of body shape dissatisfaction (BSQ), indicated by values above the cut-off of 100, was 6.6% in the second trimester, 2.9% in the third trimester, 11.0% three months, and 10.3% six months postpartum (Table 2). The proportion of women reporting elevated levels of body image dissatisfaction at least at one measurement point was 7.3% (n = 10) during pregnancy, 12.5% (n = 17) in the postpartum period, and 15.4% (n = 21) during pregnancy or postpartum. The proportion of women reporting elevated levels of body image dissatisfaction at each of the four measurement points was 2.2% (n = 3). Across all measurement points, the most frequently occurring pattern was having elevated levels of body image dissatisfaction both three and six months postpartum (n = 7, 5.2%).

**Table 2:** Trajectory of body image dissatisfaction during pregnancy and postpartum.

	T0 (pre-pregnancy)	T1 (second trimester)^a^	T2 (thirdtrimester)^b^	T3 (3 months postpartum)	T4 (6 months postpartum)	Results of repeated measures ANOVA		
						Time effect	p-value (η^2^)	Significant Post-hoc comparisons^c^
BSQ, *M* (*SD*)		59.07 (21.89)	55.51 (19.56)	66.52 (26.08)	64.15 (26.85)	F(2.38,321.81) = 23.23	**< .001** (0.15)	T1 > T2 T1 < T3, T4 T2 < T3, T4
BSQ > 100, *n* (%)		9 (6.6)	4 (2.9)	15 (11.0)	14 (10.3)	Q(3) = 14.90	**.002**	T2 < T3, T4
EDE-Q Weight, *M* (*SD*)	1.10 (1.04)	0.58 (0.76)	0.59 (0.83)	1.20 (1.17)	1.12 (1.18)	F(3.12,428.68) = 29.14	**< .001** (0.18)	T0 > T1, T2 T1 < T3, T4 T2 < T3, T4
EDE-Q Shape Concern, *M* (*SD*)	1.41 (1.15)	0.80 (0.88)	0.80 (0.88)	1.68 (1.41)	1.49 (1.34)	F(3.02,407.12) = 47.59	**< .001** (0.26)	T0 > T1, T2 T0 < T3 T1 < T3, T4 T2 < T3, T4

Notes. BSQ Body Shape Questionnaire, EDE-Q Eating Disorder Examination-Questionnaire, Q Cochran`s Q-test; η^2^ partial Eta Quadrat;

^**a**^ 18th-22nd week of gestation,

^**b**^ 33^rd^ -37th week of gestation,

^**c**^ Bonferroni post-hoc comparison.

### The trajectory of body image dissatisfaction during pregnancy and postpartum

Descriptive characteristics and the results of the repeated measures ANOVA for each of the body image dissatisfaction outcomes from pre-pregnancy to six months postpartum are presented in Table 2. Overall, the time effect was significant for the three body image dissatisfaction outcomes, with large effect sizes ranging from η^2^ = .15 (BSQ) to.26 (EDE-Q Shape Concern), indicating a significant change in body image dissatisfaction throughout pregnancy and postpartum.

Concerning the BSQ sum score, posthoc tests revealed that *body shape dissatisfaction* was significantly larger in the second compared to the third trimester (*p* = .02, *d = 0*.*26*), that it was significantly lower in the second trimester compared to three and six months postpartum (*p* < .001, *d = -0*.*40*; *p* = .02, *d = -0*.*26*) and also significantly lower in the third trimester compared to three and six months postpartum (*p* < .001, *d = -0*.*63*; *p* < .001, *d = -0*.*45*). The proportion of women suffering from elevated levels of body image dissatisfaction was significantly lower in the third trimester compared to three and six months postpartum [2.9% vs. 11.0% and 10.3%; z = -3.42, *p* = .004; z = -3.11, *p* = .01]. In summary, the data reveals a pattern where body shape dissatisfaction decreases from the second to the third trimester, increases again after childbirth, and then remains stable up to six months postpartum.

Concerning the EDE-Q, posthoc tests revealed that *Weight Concern* was significantly larger before pregnancy as compared to the second and third trimesters (*p* < .001, *d = 0*.*70*; *p* < .001, *d = 0*.*51*), that it was significantly lower in the second trimester compared to three and six months postpartum (*p* < .001, *d = -0*.*63*; *p* < .001, *d = -0*.*55*), and also significantly lower in the third trimester compared to three and six months postpartum (*p* < .001, *d = -0*.*64*; *p* < .001, *d = -0*.*52*). In summary, the data indicates the following pattern: Weight Concern decreases from pre-pregnancy levels to the second trimester, remains stable at a low level through the third trimester, increases significantly postpartum, and then remains stable up to six months after birth.

Post-hoc tests revealed that *Shape Concern* was significantly larger before pregnancy compared to the second and third trimesters (*p* < .001, *d = 0*.*78*; *p* < .001, *d = 0*.*59*), that it was significantly lower before pregnancy compared to three months postpartum (*p* = .04, *d* = .*25*), that it was significantly lower in the second trimester compared to three and six months postpartum (*p* < .001, *d = -0*.*80*; *p* < .001, *d = -0*.*64*), and also significantly lower in the third trimester compared to three and six months postpartum (*p* < .001, *d = -0*.*81*; *p* < .001, *d = -0*.*66*). In summary, the data indicates the following pattern: Shape Concern decreases from pre-pregnancy levels to the second trimester, remains stable at a low level through the third trimester, increases above pre-pregnancy levels three months postpartum, and remains relatively stable up to six months after birth.

Overall, the most prominent effect sizes (Cohen’s d) for mean differences between two measurement points were found for the decrease of body image dissatisfaction from pre-pregnancy to the second trimester of pregnancy and the increase of body image dissatisfaction from the third trimester to three and six months postpartum. This finding was particularly evident with the EDE-Q Shape Concern subscale.

### The trajectory of body image dissatisfaction during pregnancy and postpartum in relationship to pre-pregnancy BMI

**Fig 2:**
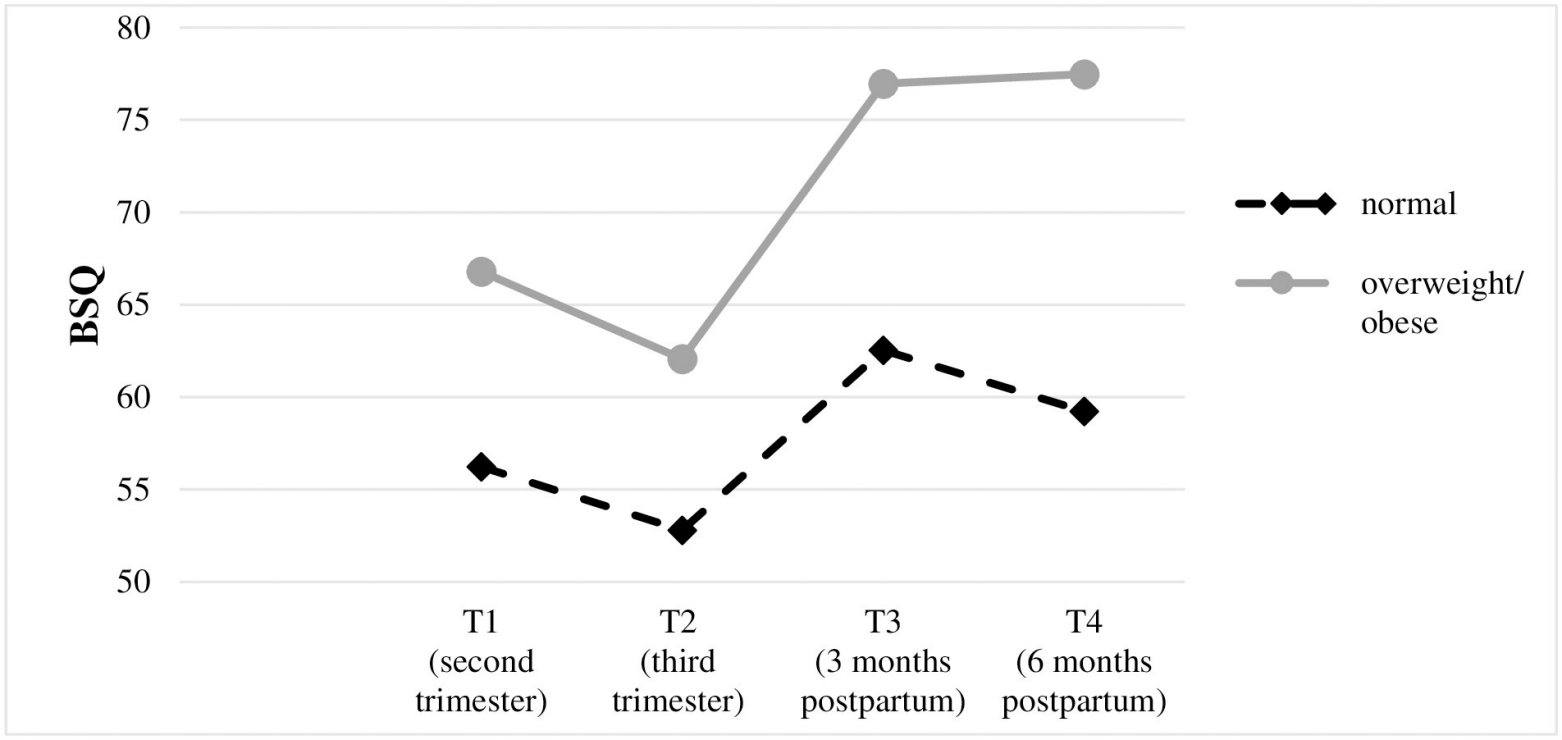
Trajectories of body shape dissatisfaction (mean) in relationship to BMI group.

**Fig 3:**
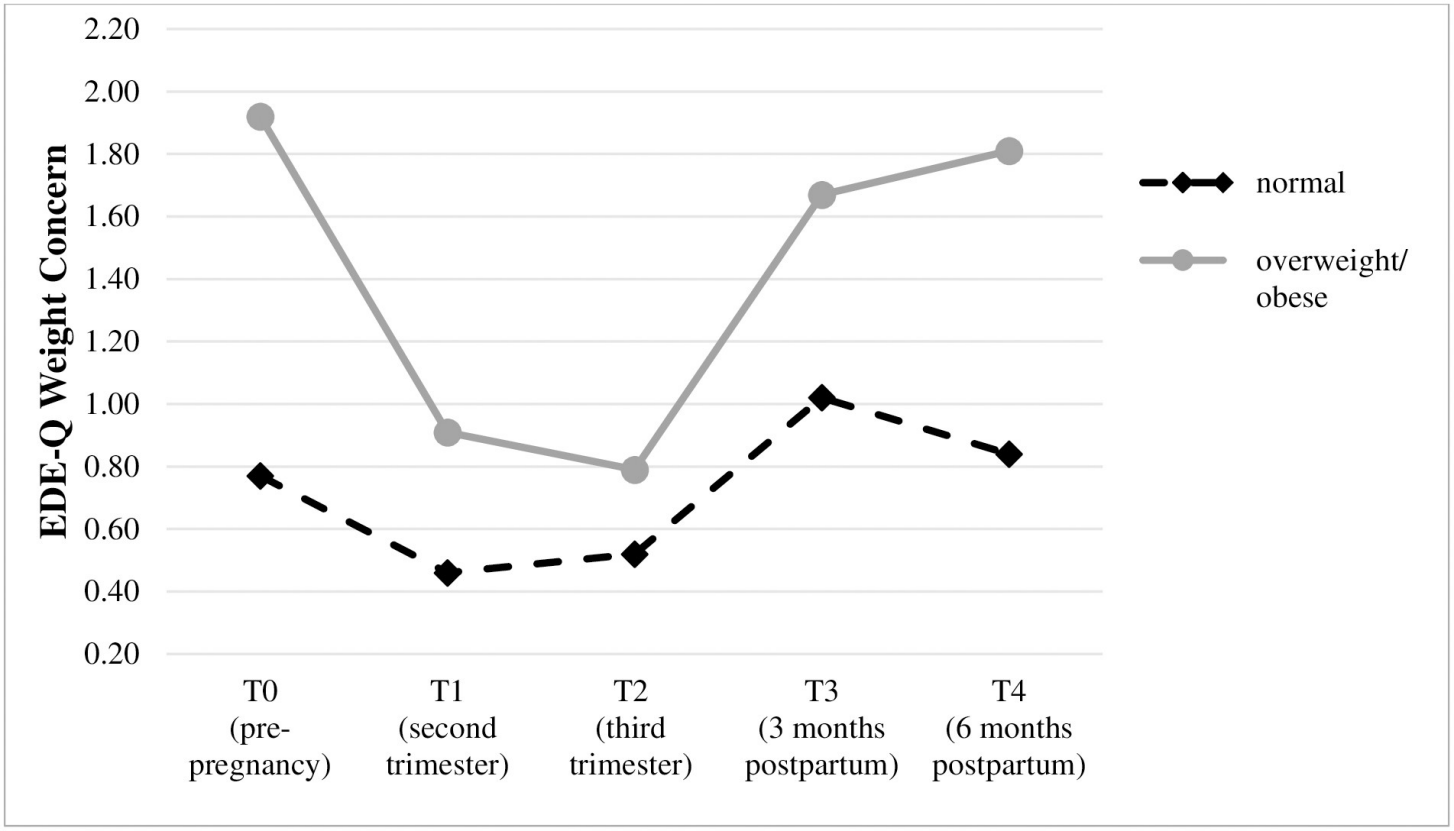
Trajectories of weight concerns (mean) in relationship to BMI group.

**Fig 4:**
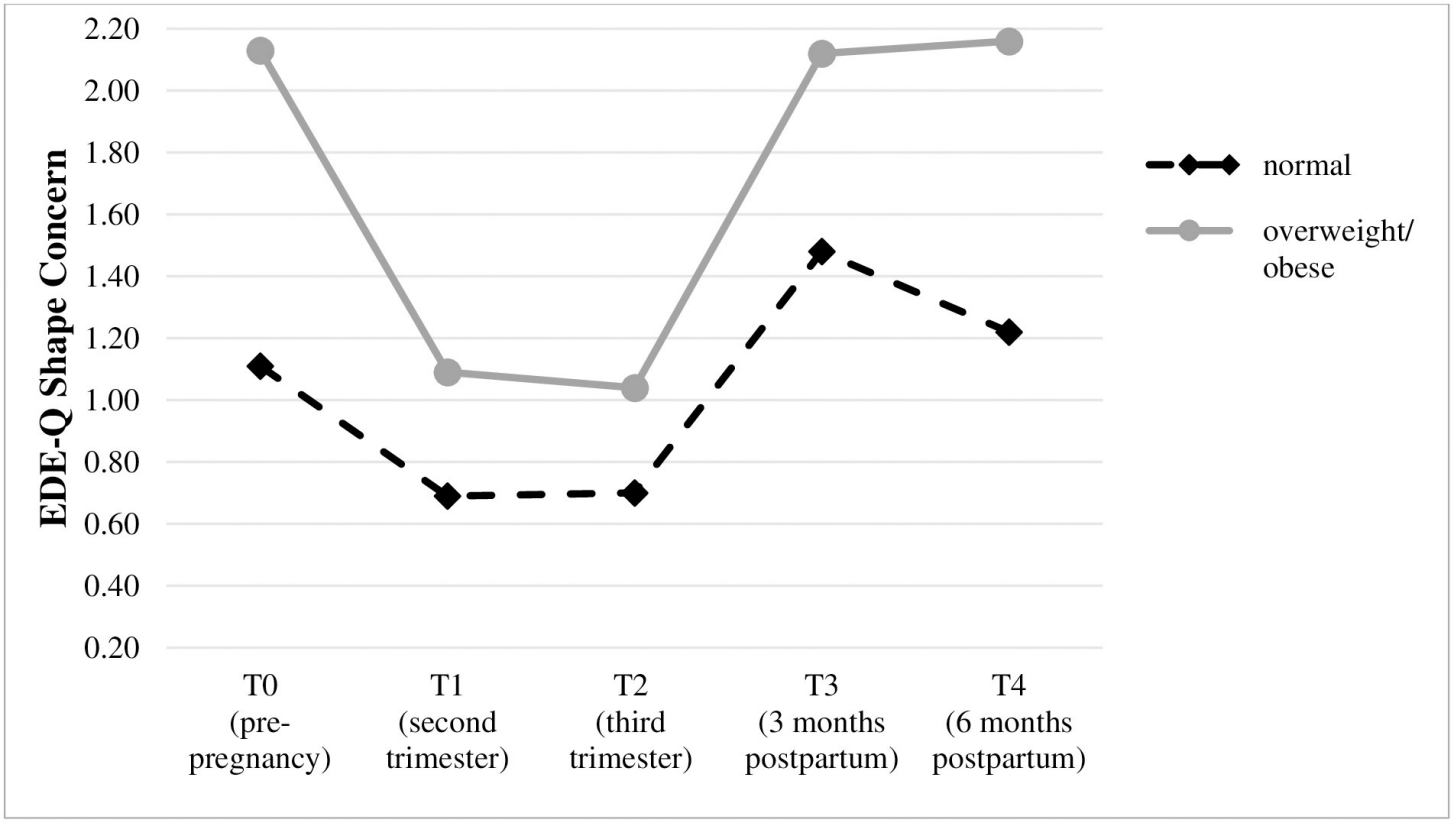
Trajectories of shape concerns (mean) in relationship to BMI group.

Due to the small number of cases in the obese category (n = 15), the overweight and obese subgroups were combined [overweight/obese (n = 41); normal weight (n = 90)] in order to provide sufficient statistical power. Table 3 presents descriptive characteristics and the results of the mixed between-within ANOVA for each of the body image dissatisfaction outcomes from pre-pregnancy to six months postpartum in relationship to pre-pregnancy BMI (normal weight vs. overweight/obese). The course of body image dissatisfaction in relationship to women’s BMI for each of the three measures are also displayed in Figs 2–4.

**Table 3:** Trajectory of body image dissatisfaction during pregnancy and postpartum in relationship to pre-pregnancy BMI.

		T0 (pre-pregnancy)	T1 (second trimester)^a^	T2 (third trimester)^b^	T3 (3 months postpartum)	T4 (6 months postpartum)	Results of the mixed ANOVA			
							Main effect group Main effect time Interaction effect	p-value (η^2^)	Simple main effects of time	Simple main effects of group
BSQ, *M* (*SD*)	normal		56.24 (19.76)	52.80 (17.91)	62.54 (22.24)	59.23 (21.33)	F(1,129) = 11.28 F(2.41,310.86) = 26.40 F(2.41,310.86) = 3.23	**.001** (.08) **< .001** (.17) **.03** (.02)	T1 < T3 T2 < T3, T4	normal < overweight/obese for T1, T2, T3, T4
	overweight/obese		66.79 (25.20)	62.07 (22.08)	76.96 (31.63)	77.48 (33.47)			T1 < T3, T4 T2 < T3, T4	
EDE-Q Weight, *M* (*SD*)	normal	0.77 (0.72)	0.46 (0.61)	0.52 (0.73)	1.02 (1.02)	0.84 (0.88)	F(1,129) = 23.31 F(3.15,210.92) = 36.93 F(3.15,210.92) = 9.10	**< .001** (.15) **< .001** (.22) **< .001** (.07)	T0 > T1 T1 < T3, T4 T2 < T3, T4	normal < overweight/obese for T0, T1, T3, T4
	overweight/obese	1.92 (1.18)	0.91 (0.98)	0.79 (1.03)	1.67 (1.39)	1.81 (1.48)			T0 > T1, T2 T1 < T3, T4 T2 < T3, T4	
EDE-Q Shape Concern, *M* (*SD*)	normal	1.11 (0.90)	0.69 (0.79)	0.70 (0.80)	1.48 (1.25)	1.22 (1.06)	F(1,129) = 14.98 F(3.02,389.09) = 51.44 F(3.02,389.09) = 5.76	**< .001** (.10) **< .001** (.29) **< .001** (.04)	T0 > T1, T2 T0 < T3 T1 < T3, T4 T2 < T3, T4 T3 > T4	normal < overweight/obese for T0, T1, T3, T4
	overweight/obese	2.13 (1.35)	1.09 (1.02)	1.04 (1.05)	2.19 (1.65)	2.16 (1.69)			T0 > T1, T2 T1 < T3, T4 T2 < T3, T4	

Notes. BSQ Body Shape Questionnaire, EDE-Q Eating Disorder Examination-Questionnaire; η^2^ partial Eta Quadrat;

^**a**^ 18th-22nd week of gestation,

^**b**^ 33 ^rd^-37th week of gestation.

Overall, the main effects of group and time and the group x time interaction effect were significant for each of the three body image dissatisfaction outcomes. Concerning the main effect of group, effect sizes ranged from η^2^ = .08 (BSQ) to.15 (EDE-Q Weight Concern), indicating moderate to large mean differences between the normal weight and overweight/obese group. Concerning the main effect of time, effect sizes ranged from η^2^ = .17 (BSQ) to.29 (EDE-Q Shape Concern), indicating large mean differences for body image dissatisfaction over time. Regarding the group x time interaction effect, effect sizes ranged from small to moderate with η^2^ = .02 (BSQ) to.07 (EDE-Q Weight Concern), indicating that the change in body image dissatisfaction depended at least for a small to moderate extent on BMI. Therefore, simple main effects of group and time (see last column of Table 3) and post hoc comparisons were analyzed separately for each of the three body image dissatisfaction measures. The results of these analyses are described in the next sections.

Concerning the BSQ sum score, *body shape dissatisfaction* was significantly lower in the normal weight compared to the overweight/obese group in the second [t(129) = -2.60, p = .01, d = -0.49] and third trimester [t(129) = -2.55, p = .01, d = -0.48] as well as three [t(58.70) = -2.64, p = .01, d = -0.57] and six months postpartum [t(55.32) = -3.21, p < .001, d = -0.71]. Within the normal weight and the overweight/obese group, a significant effect of time on the BSQ sum score was found [F(2.53,225.14) = 12.95, p < .001, η^2^ = .13; F(2.03,81.30) = 12.20, p < .001, η^2^ = .23]. Within the normal weight group, posthoc tests revealed that body image dissatisfaction was significantly lower in the second trimester compared to three months postpartum (*p* = .01, *d = -0*.*36*) and also significantly lower in the third trimester compared to three and six months postpartum (*p* < .001, *d = -0*.*59*; *p* = .001, *d = -0*.*41*). Within the overweight/obese group, posthoc tests revealed that body image dissatisfaction was significantly lower in the second trimester compared to three and six months postpartum (*p* = .03, *d = -0*.*46; p* = .04, *d = -0*.*44*) and also significantly lower in the third trimester compared to three and six months postpartum (*p* < .001, *d = -0*.*75*; *p* = .001, *d = -0*.*71*).

Concerning the EDE-Q, *Weight Concern* was significantly lower in the normal weight compared to the overweight/obese group before pregnancy [t(53.90) = -5.76, p < .001, d = -1.29], in the second trimester [t(54.66) = -2.72, p = .01, d = -0.61], three months [t(60.49) = -2.65, p = .01, d = -0.56] and six months postpartum [t(53.28) = -3.90, p < .001, d = -0.88]. In the third trimester, no significant mean differences were found. Within the normal weight and the overweight/obese group, a significant effect of time on the EDE-Q Weight Concern subscale was found [F(3.28,292.26) = 13.49, p < .001, η^2^ = .13; F(2.45,98.16) = 22.18, p < .001, η^2^ = .36]. Within the normal weight group, posthoc tests revealed that weight concern was significantly higher before pregnancy compared to the second trimester (p < .001, *d = 0*.*49*), and it was significantly lower in the second trimester compared to three and six months postpartum (*p* < .001, *d = -0*.*62; p* < .001, *d = -0*.*45*) and also significantly lower in the third trimester compared to three and six months postpartum (*p* < .001, *d = -0*.*55*; *p* = .02, *d = -0*.*35*). Within the overweight/obese group, posthoc tests revealed that weight concern was significantly higher before pregnancy compared to the second and third trimesters (p < .001, *d = 1*.*31*; p < .001, *d = 1*.*17*), and it was significantly lower in the second trimester compared to three and six months postpartum (*p* = .002, *d = -0*.*64; p* < .001, *d = -0*.*74*) and also significantly lower in the third trimester compared to three and six months postpartum (*p* < .001, *d = -0*.*89*; *p* = .02, *d = -0*.*93*).

*Shape Concern* was significantly lower in the normal weight compared to the overweight/obese group before pregnancy [t(56.70) = -4.41, p < .001, d = -0.96], in the second trimester [t(63.00) = -2.21, p = .03, d = -0.46], three months [t(61.85) = -2.46, p = .02, d = -0.51] and six months postpartum [t(54.85) = -3.28, p = .002, d = -0.73]. In the third trimester, no significant mean differences were found. Within the normal weight and the overweight/obese group, a significant effect of time on the EDE-Q Shape Concern subscale was found [F(3.01,267.93) = 25.76, p < .001, η^2^ = .22; F(2.62,104.67) = 24.10, p < .001, η^2^ = .38]. Within the normal weight group, posthoc tests revealed that shape concern was significantly higher before pregnancy compared to the second and third trimesters (p < .001, *d = 0*.*63*; p < .001, *d = 0*.*44*), that it was significantly lower before pregnancy compared to three months postpartum (p = .01, *d = -0*.*36*), significantly lower in the second trimester compared to three and six months postpartum (*p* < .001, *d = -0*.*77; p* < .001, *d = -0*.*58*), significantly lower in the third trimester compared to three and six months postpartum (*p* < .001, *d = -0*.*72*; *p* = .02, *d = -0*.*57*) and significantly higher three months compared to six months postpartum (*p* = .02, *d = 0*.*33*). Within the overweight/obese group, posthoc tests revealed that shape concern was significantly higher before pregnancy compared to the second and third trimesters (p < .001, *d = 1*.*23;* p < .001, *d = 0*.*97*), and it was significantly lower in the second trimester compared to three and six months postpartum (*p* < .001, *d = -0*.*88; p* < .001, *d = -0*.*80*) and also significantly lower in the third trimester compared to three and six months postpartum (*p* < .001, *d = -1*.*05 p* < .001, *d = -0*.*90*).

## Discussion and conclusion

This prospective longitudinal study aimed to investigate the frequency and change in body image dissatisfaction during pregnancy and postpartum and its relationship to pre-pregnancy BMI. The accruing sample consisted of healthy pregnant women recruited while waiting for routine prenatal diagnostic appointments at a university hospital in a major city. The sample resembles the general German population in terms of essential characteristics (average age, BMI). However, household net income was below the average [45–47].

**Regarding the first aim**, the results show that prevalence rates of women suffering from body shape dissatisfaction ranged between 2.9 and 6.6% during pregnancy and 10.3 to 11.0% postpartum. 15.4% of women reported high body shape dissatisfaction at least once, but only 2.2% experienced body shape dissatisfaction consistently during pregnancy and postpartum. Overall, this study’s proportion of women with high body shape dissatisfaction during pregnancy is much lower than previously reported [34–45% [7,8]]. In previous studies, the use of self-developed single items [8] and unspecified cut-off values [7] might account for these differences. Using a validated questionnaire with existing cut-off values in the current study increases confidence in the results. Furthermore, to our knowledge, this is the first study reporting prevalence rates in the postpartum period. Notably, women reporting higher levels of body image dissatisfaction at T1 had a significantly higher risk of study drop-out, which might have led to an underestimation of the proportion of women suffering from body shape dissatisfaction, particularly postpartum.

The average appraisal of body shape dissatisfaction (mean value of the BSQ) in the second trimester and postpartum was comparable to those of a representative sample of non-pregnant women between 25–34 years living in Germany [40]. However, body shape dissatisfaction during the third trimester was lower compared to a representative sample, implying that particularly during the last trimester of pregnancy, preoccupation with the body’s appearance is reduced, and its function and capacity might be more in focus. The average appraisal of weight and shape concern (mean values of subscales of the EDE-Q) of the current sample was lower than in an accruing predominantly female sample recruited at German universities [41]. The finding of lower levels of body image dissatisfaction in pregnant women as compared to non-pregnant women might support the theory that women experience liberation from social ideals during pregnancy. As comparisons are only made on a descriptive level, this interpretation must be verified by studies directly comparing body image dissatisfaction between pregnant and non-pregnant women. In a recent meta-analysis [4] based on 17 studies with cross-sectional, longitudinal, or retrospective designs, no significant differences in body image dissatisfaction between pregnant and non-pregnant women were found. Eight studies found that body image dissatisfaction was lower in pregnant women, five found the opposite, and five found no significant group differences.

**Regarding the second aim,** findings show that the extent of body dissatisfaction decreased from the time before pregnancy to the second trimester, mainly remained stable from the second to the third trimesters (EDE-Q weight and shape concern), or decreased even more (BSQ), increased again to pre-pregnancy level or even beyond (EDE-Q shape concern) three-month postpartum and mainly remained stable until six months postpartum. Furthermore, results show that the course of body image dissatisfaction during pregnancy and postpartum was comparable for the three measures considered the gold standard for measuring body image [38]. The results are in line with other findings [12–16] that showed that body image dissatisfaction was reduced during pregnancy but reached pre-pregnancy levels again three months after delivery. According to Davies & Wardle (1994), it could be hypothesized that culturally defined beauty ideals might be less relevant during pregnancy. In contrast, the capacity and functionality of women’s bodies are more relevant. This assumption is supported by other studies showing that preoccupation with appearance and the prioritization of appearance over function is reduced over pregnancy [33]. Postpartum, the social pressure to be thin and in good shape, which is communicated via media and at least partly mediated by thin-ideal internalization and social comparison [48], might be one reason for the renewed increase of body image dissatisfaction in all three measures. The decrease within pregnancy and the increase postpartum was particularly pronounced for shape concern and less strong for weight concern and overall body shape dissatisfaction, as indicated by large compared to moderate effect sizes. This finding implies that the adaption to changes in body shape is more complicated than to changes in body weight three months postpartum.

The significant and sustained increase in body image dissatisfaction postpartum, potentially affecting around 10% of women, underscores the need for targeted support, especially during this time. Therefore, postpartum interventions aimed at addressing body image concerns could be particularly beneficial. Based on results of a meta-analysis [49], effective interventions to improve body image should include cognitive-behavioral techniques (provide size-estimate exercises, discuss cognitions and their role in body image, teach self-monitoring and restructuring of cognitions, change negative body language, conduct exposure exercises, provide stress management training and relapse-prevention strategies) and/or psychoeducation (discuss the concept of body image, discuss the behavioural expression, the consequences and causes of negative body image). Additionally, incorporating self-compassion and cognitive reappraisal tailored to new mothers could help mitigate body image dissatisfaction [50,51].

**Regarding the third aim**, the results demonstrate that women with overweight or obesity before pregnancy experienced higher levels of body image dissatisfaction before pregnancy, in the second trimester, and three and six months postpartum than women within the normal weight range. This finding was consistent for all three body image measures. These results are in line with previous studies [3,6,13,32] and underline the relevance of pre-pregnancy BMI as a risk factor for body image dissatisfaction during pregnancy. The results extend previous results by showing that a higher pre-pregnancy BMI was also a risk factor for postpartum body dissatisfaction at three and six months postpartum. Overall, the mean differences in body dissatisfaction for all three measures between women with overweight or obesity before pregnancy and women within the normal weight group were moderate to large, with overweight or obese women being more dissatisfied. In addition, mean differences were larger before and after pregnancy than within pregnancy. Interestingly, mean differences were non-significant within the third trimester of pregnancy for weight and shape concerns. This finding might imply that pre-pregnancy BMI loses some impact in the last part of pregnancy, when gestational weight gain peaks, and weight and shape differences might be less relevant for women than the functionality of the body and its capacity to create new life. Furthermore, the current results indicate that women with overweight or obesity before pregnancy start at a higher level of body image dissatisfaction before pregnancy but show a similar trajectory of body image dissatisfaction over pregnancy and postpartum as women within the normal weight range for each of the three body image measures.

Besides these similarities, the significant interaction effect indicates some relevant differences between BMI groups. First, women with overweight or obesity before pregnancy showed a stronger decline during pregnancy and a stronger increase in body image dissatisfaction after pregnancy (as indicated by effect sizes), implying that changes in body image dissatisfaction are more pronounced within women with overweight or obesity compared to women within the normal weight group. Second, results suggest that within the overweight/obese group, body shape dissatisfaction six months after childbirth (as indicated by the BSQ) does not decline to the second-trimester level but remains stable from three to six months postpartum. In contrast, within the normal weight group, a decline was noticeable. This finding might imply that women with overweight or obesity before pregnancy have more difficulties adapting to postpartum body appearance. Third, within the overweight/obese group, pre-pregnancy weight concerns were larger than third-trimester weight concerns, whereas, in the normal weight group, this was not the case. Furthermore, at least on a descriptive level, weight concerns decline from the second to the third trimester in the overweight/obese group, whereas in the normal weight group, they tend to increase at the end of pregnancy. This finding might imply that within the overweight/obese group, weight concerns start to increase later compared to the normal weight group. Fourth, within the normal weight group, shape concerns three months after childbirth were larger than before pregnancy and larger than six months after childbirth. In contrast, no significant differences were found in the overweight/obese group. These results might indicate that women in the normal weight range had more difficulties adapting to remaining postpartum shape differences and needed more time to reach pre-pregnancy levels. However, since the effect size for the interaction effect is smaller than the main effects of time and BMI and the assumption of the mixed effects ANOVA was partly not met, these interaction effects must be interpreted cautiously.

Since a higher BMI has been identified as a risk factor for body image dissatisfaction, pregnant women and young mothers should be informed about and encouraged, to participate in effective intervention programs aimed at reducing BMI, such as those involving exercise or dietary treatment [52–54].

### Strengths and limitations

The study`s strengths were the prospective study design, which included six months postpartum, using two standardized questionnaires, considered the gold standard for measuring body image, and a sample resembling the general population in essential characteristics. The first limitation relates to the relatively high number of eligible pregnant women who did not participate in our study (see Fig 1). There was no information about the reasons for non-participating. However, it can be assumed that language barriers, comprehension difficulties in the informed consent process, lack of time, and lack of interest in the study might be possible reasons. Besides this, recruiting women immediately before their routine prenatal diagnostic appointment might not have been ideal, as many women were more or less in a tense and anxious mood because they were not yet sure whether their baby was healthy. In addition, after study entry, many women dropped out, in particular after childbirth. Most women provided no information about the reasons for quitting study participation, as seen in Fig 1. It can be hypothesized that women had less time after childbirth and that they focused on nursing the child, dealing with hormonal and physical changes, planning to return to work, and adapting to the new role as a mother. High attrition rates are not uncommon in longitudinal studies with a sample of pregnant women. In a review [55] of studies investigating ante- and postpartum depression in women, one-quarter of studies reported attrition rates between 37.3 and 49.9%. In the current study, women with more substantial body image dissatisfaction during pregnancy had a higher risk of study drop-out. It can be hypothesized that these women felt uncomfortable when they were asked to report on the degree of body image dissatisfaction and body weight after childbirth and, therefore, terminated study participation. This might have led to an underestimation of prevalence rates and bias in the course of body image dissatisfaction, mainly three and six months postpartum. The second limitation relates to the retrospective measurement of weight and EDE-Q weight and shape concern before pregnancy, which might have led to a biased estimation of BMI and body image before pregnancy. However, women reported high confidence levels in their body weight assessment, so the estimates could be considered reliable. Furthermore, despite some differences between self-reported and measured pre-pregnancy BMI, the association with perinatal outcomes might be similar [56]. Assessing women before and during the first trimester of pregnancy would be beneficial. However, this remains difficult to implement, as many women do not know when they will get pregnant. Third, violations of the prerequisite for the mixed between-within ANOVA might have led to a higher probability of committing a type I error concerning the influence of pre-pregnancy BMI on EDE-Q weight and shape concern. Future studies should verify the results in a larger sample with larger BMI subgroups, leading to estimates that are more robust and the opportunity to analyze trajectories of body image in women with overweight and obesity separately.

## Conclusions

The prevalence of body image dissatisfaction is relatively low during pregnancy but affects approximately every tenth woman postpartum. Body image dissatisfaction decreases from pre-pregnancy to pregnancy, increases three months postpartum, and returns to pre-pregnancy levels six months postpartum. A higher pre-pregnancy BMI is a risk factor for body image dissatisfaction in the second trimester and particularly postpartum, but less relevant during the third trimester. Health care providers might encourage overweight and obese women to lose weight before, during, and after pregnancy and recommend weight-management interventions, such as exercise and nutrition-based programs, which should ideally be provided free of charge. Overall, it can be concluded that pregnancy might be experienced as a period where women are released from the pressure to be thin and in shape from sociocultural and media influences. They may change their focus from appearance-related aspects of the body to the functionality of the body and its capacity to create new life. Due to the negative impact of body image dissatisfaction on maternal and child health, healthcare providers should assess, monitor, and provide support for women suffering from body image dissatisfaction postpartum, particularly women with higher pre-pregnancy BMI levels.

As the current study ends six months after childbirth, it remains unclear how body image dissatisfaction develops beyond this time. Furthermore, similarities and differences in the prevalence and course of body image dissatisfaction in pregnant and non-pregnant populations of women are not yet investigated. Therefore, future studies should include further assessment points in the postpartum period and an age-matched control group of non-pregnant women. This would allow to compare pregnant and non-pregnant women during an equal period and broaden the understanding of the impact of pregnancy on women`s body image. To enhance study participation, recruitment should ideally occur outsidethe hospital context and away from potentially stressful examination appointments. Furthermore, study information and questionnaires should be provided in multiple languages and in plain language to facilitate participation among women with diverse educational backgrounds and migration histories. Due to the small sample size in combination with the high attrition rate, it was not possible to investigate differences between women with overweight and obesity. Therefore, future studies should recruit a larger sample of pregnant women to investigate and compare the prevalence and course of body image dissatisfaction in women with overweight, obesity and ideally also underweight. Besides this, a larger sample size provides the opportunity to analyze further potential moderators like differences in body image dissatisfaction between women who were pregnant with their first child and those who were pregnant with their second child. A mixed-methods design that incorporates qualitative methods, such as focus group interviews with women, could enhance the understanding of body image concerns and changes during the peripartal period.
